# Supplementary material for: Rhosin Suppressed Tumor Cell Metastasis through Inhibition of Rho/YAP Pathway and Expression of RHAMM and CXCR4 in Melanoma and Breast Cancer Cells
Source: Biomedicines. 2021 Jan 4;9(1):35. doi: 10.3390/biomedicines9010035 (PMC7824767; doi:10.3390/biomedicines9010035)

Supplementary Figure

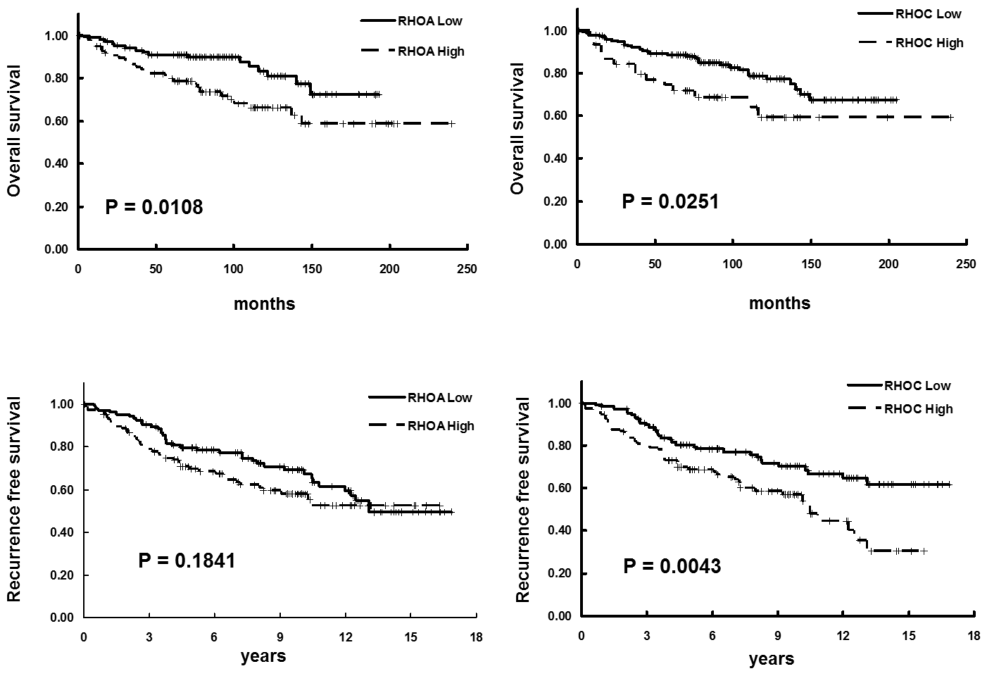

Figure S1. Overexpression of RhoA or RhoC is correlated with poor prognosis in breast cancer patients. The correlation between RhoA or RhoC expression and breast cancer patient prognosis (overall survival and recurrence free survival). The relationship between RhoA or RhoC expression and patient prognosis was examined using the SurvExpress database.

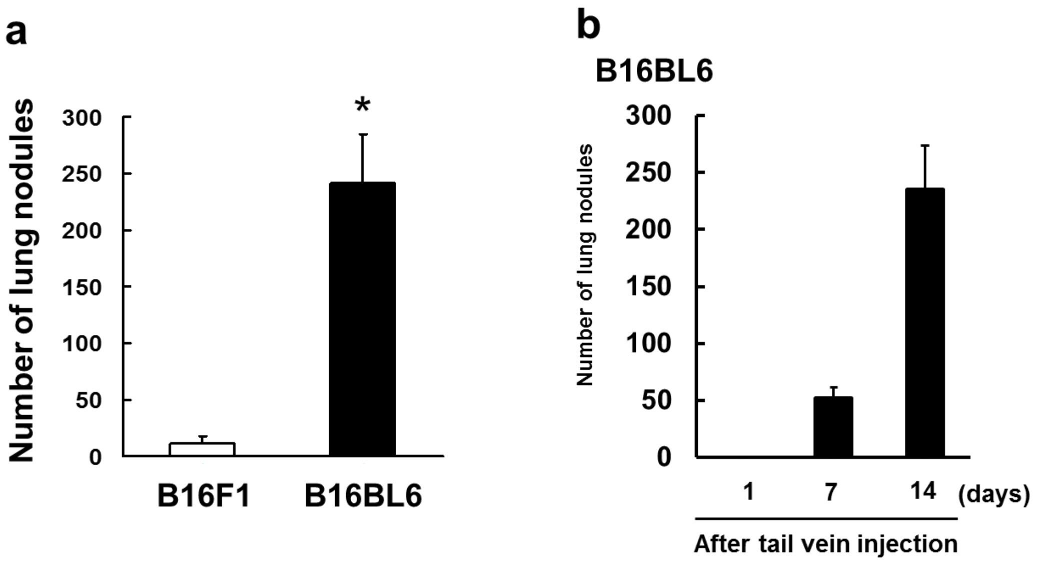

Figure S2. Confirmation of metastatic potential to B16F1 and B16BL6 cells. (a) B16F1 cells (1×10^5 cells in 0.2 ml) and B16BL6 cells (1×10^5 cells in 0.2 ml) were injected into the tail vein of syngeneic C57BL/6J mice. After 14 days, visible nodules that had metastasized to the lungs were counted. The results are expressed as the mean ± SD for 10 mice. *$p < 0.01$ vs. B16F1 cells (0.1% DMSO-treated) (ANOVA with Dunnet's test). (b) B16BL6 cells (1×10^5 cells in 0.2 ml) were injected into the tail vein of syngeneic C57BL/6J mice. After 1, 7, and 14 days, visible nodules that had metastasized to the lungs were counted. The results are expressed as the mean ± SD for 6 mice.

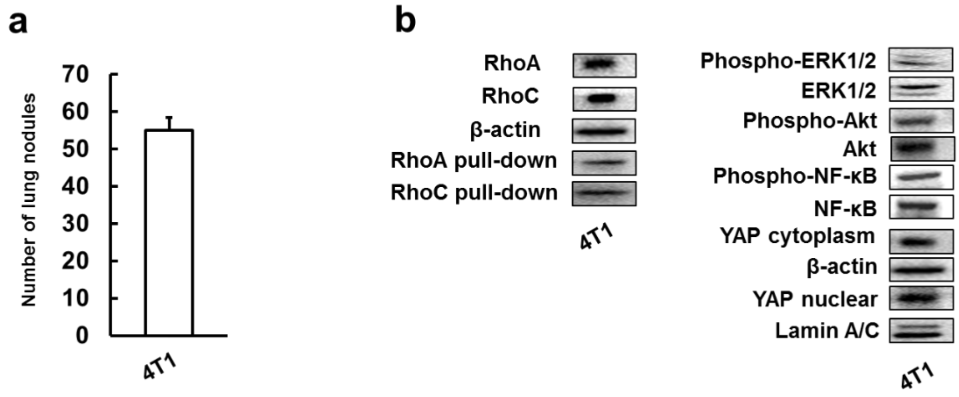

Figure S3. Confirmation of metastatic potential to 4T1 cells, expression of RhoA, RhoC, phospho-ERK1/2, phospho-Akt, phospho-NF-κB, and nuclear translocation of YAP. (a) 4T1 cells (1×10^5 cells in 0.2 ml) were injected into the tail vein of syngeneic Balb/c mice. After 14 days, visible nodules that had metastasized to the lungs were counted. The results are expressed as the mean ± SD for 10 mice. (b) Images of western blots for the RhoA, RhoC, RhoA-GTP form (RhoA pull-down), RhoC-GTP form (RhoC pull-down) and β-actin (internal standard), phospho-ERK1/2, ERK1/2, phospho-Akt, Akt, phospho-NF-κB, NF-κB, YAP, and lamin A/C.

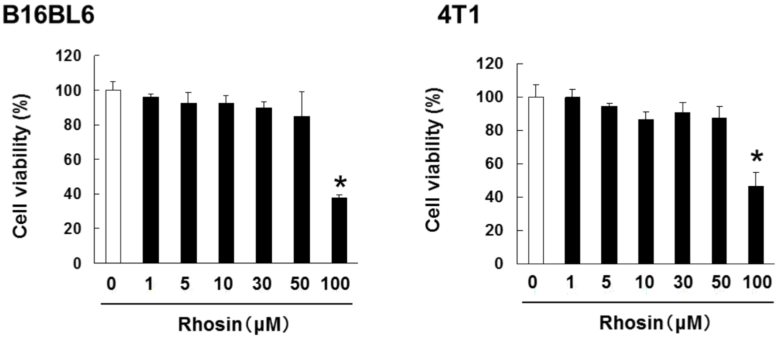

Figure S4. Determination of the rhosin concentrations suitable for administration to B16BL6 and 4T1 cells. B16BL6 or 4T1 cells were incubated in 96-well plates for 24 h and then treated with 1–100 μM rhosin. Trypan blue exclusion assay was performed after five days. The results are representative of five independent experiments. *$p < 0.01$ vs. control (ANOVA with Dunnett's test).

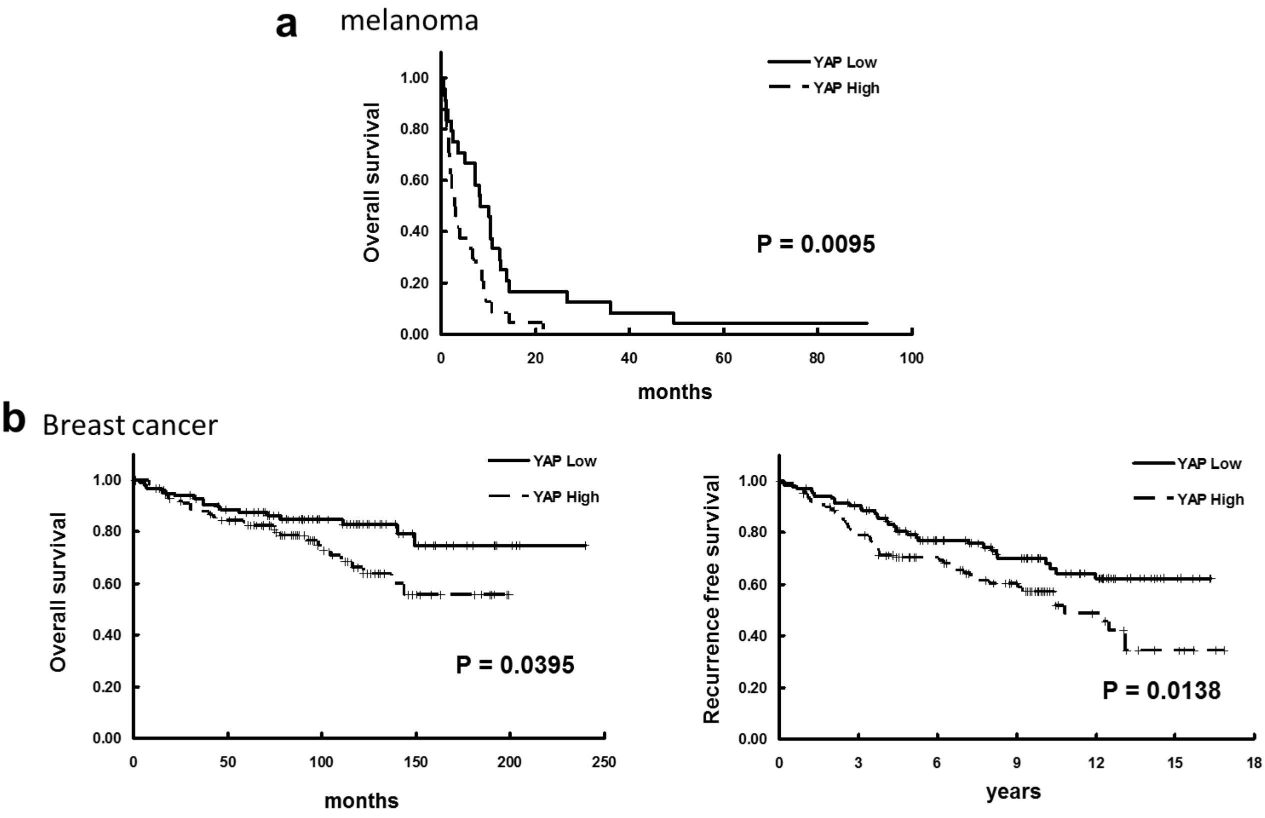

Figure S5. Overexpression of YAP is correlated with poor prognosis in melanoma and breast cancer patients. The correlation between YAP expression and (a) melanoma patient prognosis (overall survival) or (b) breast cancer prognosis (overall survival and recurrence free survival). The relationship between YAP expression and patient prognosis was examined using the SurvExpress database.

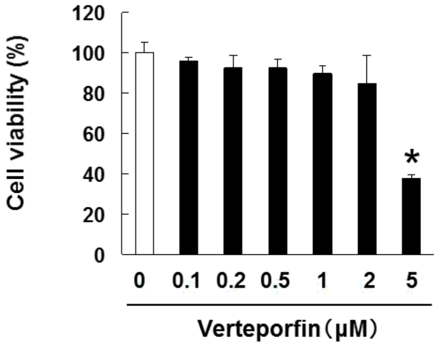

Figure S6. Determination of the verteporfin concentrations suitable for administration to B16BL6 cells. B16BL6 cells were incubated in 96-well plates for 24 h and then treated with 0.1–5 μM verteporfin. Trypan blue exclusion assay was performed after five days. The results are representative of five independent experiments. *p < 0.01 vs. control (ANOVA with Dunnett's test).

Figure 1a

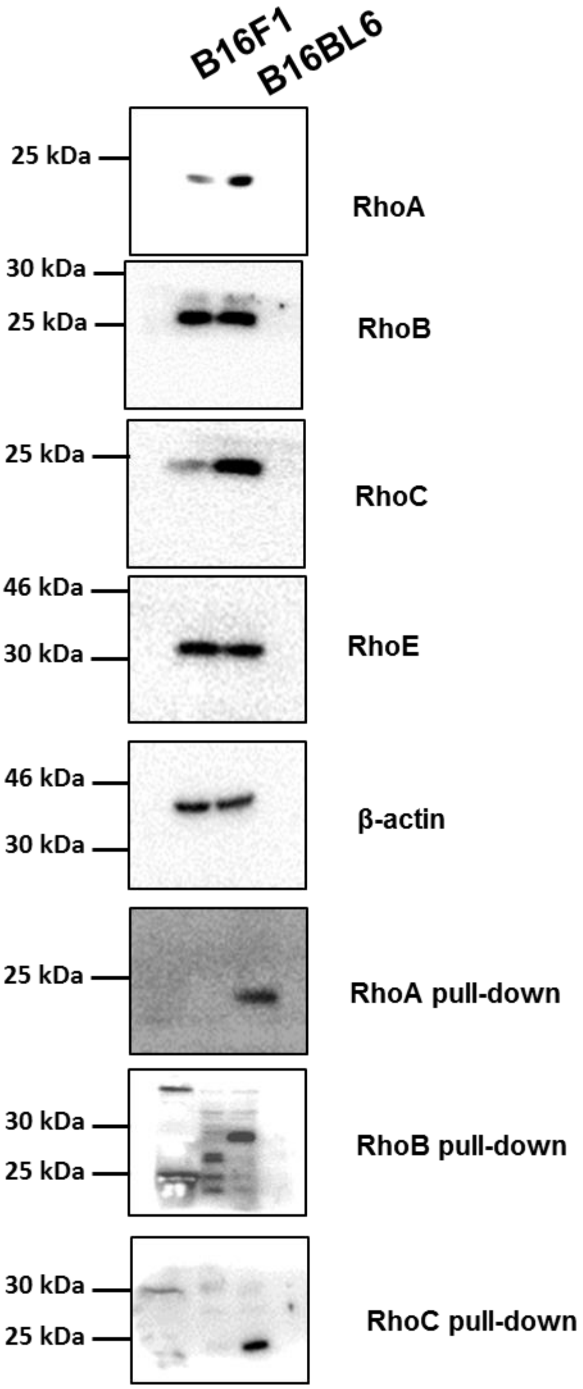
B16F1
B16BL6
25 kDa
RhoA
30 kDa
25 kDa
RhoB
25 kDa
RhoC
46 kDa
30 kDa
RhoE
46 kDa
30 kDa
β-actin
25 kDa
RhoA pull-down
30 kDa
25 kDa
RhoB pull-down
30 kDa
25 kDa
RhoC pull-down

Figure 1b

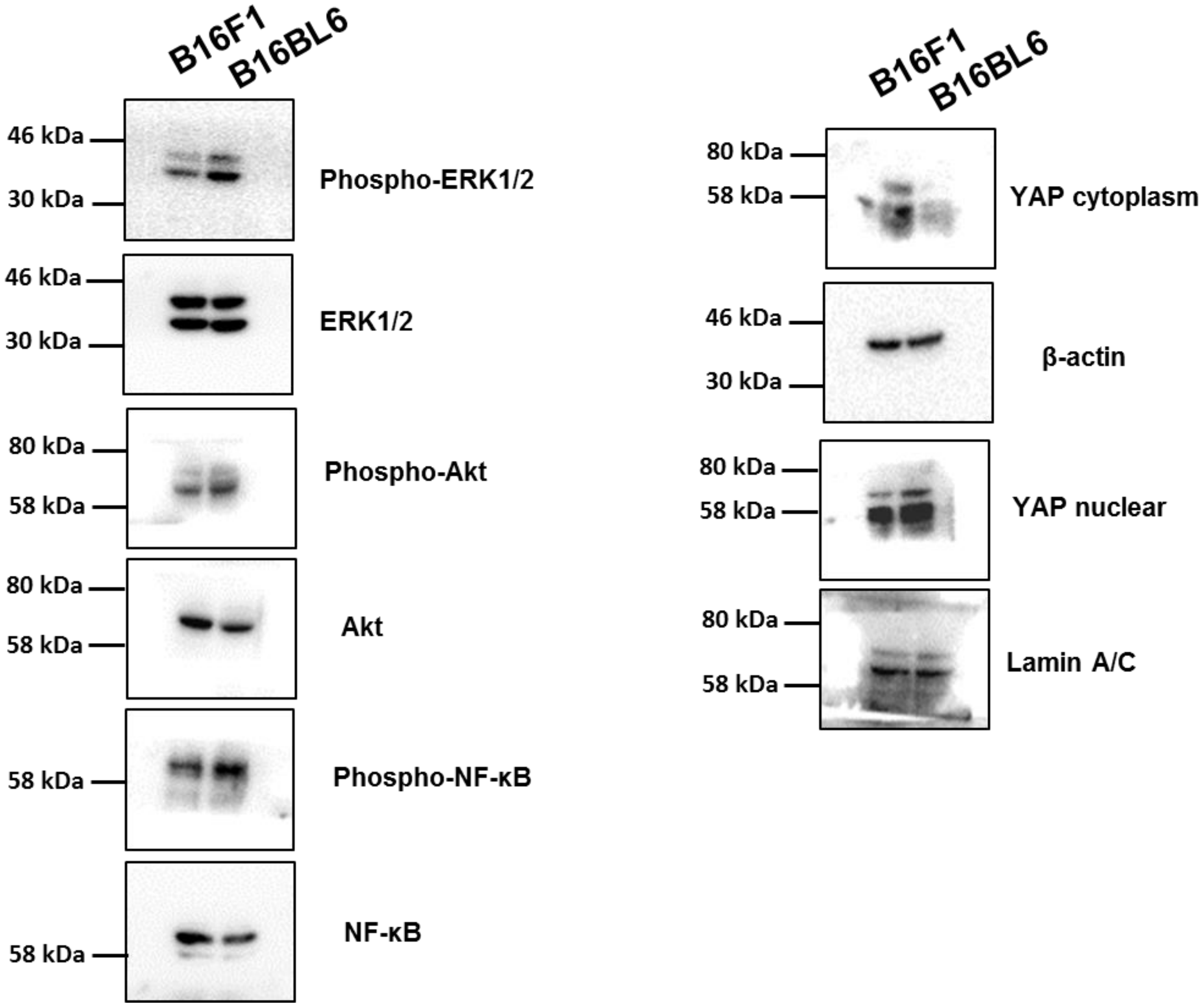
B16F1
B16BL6
46 kDa
30 kDa
Phospho-ERK1/2
46 kDa
30 kDa
ERK1/2
80 kDa
58 kDa
Phospho-Akt
80 kDa
58 kDa
Akt
58 kDa
Phospho-NF-κB
58 kDa
NF-κB
B16F1
B16BL6
80 kDa
58 kDa
YAP cytoplasm
46 kDa
30 kDa
β-actin
80 kDa
58 kDa
YAP nuclear
80 kDa
58 kDa
Lamin A/C

Figure 2a

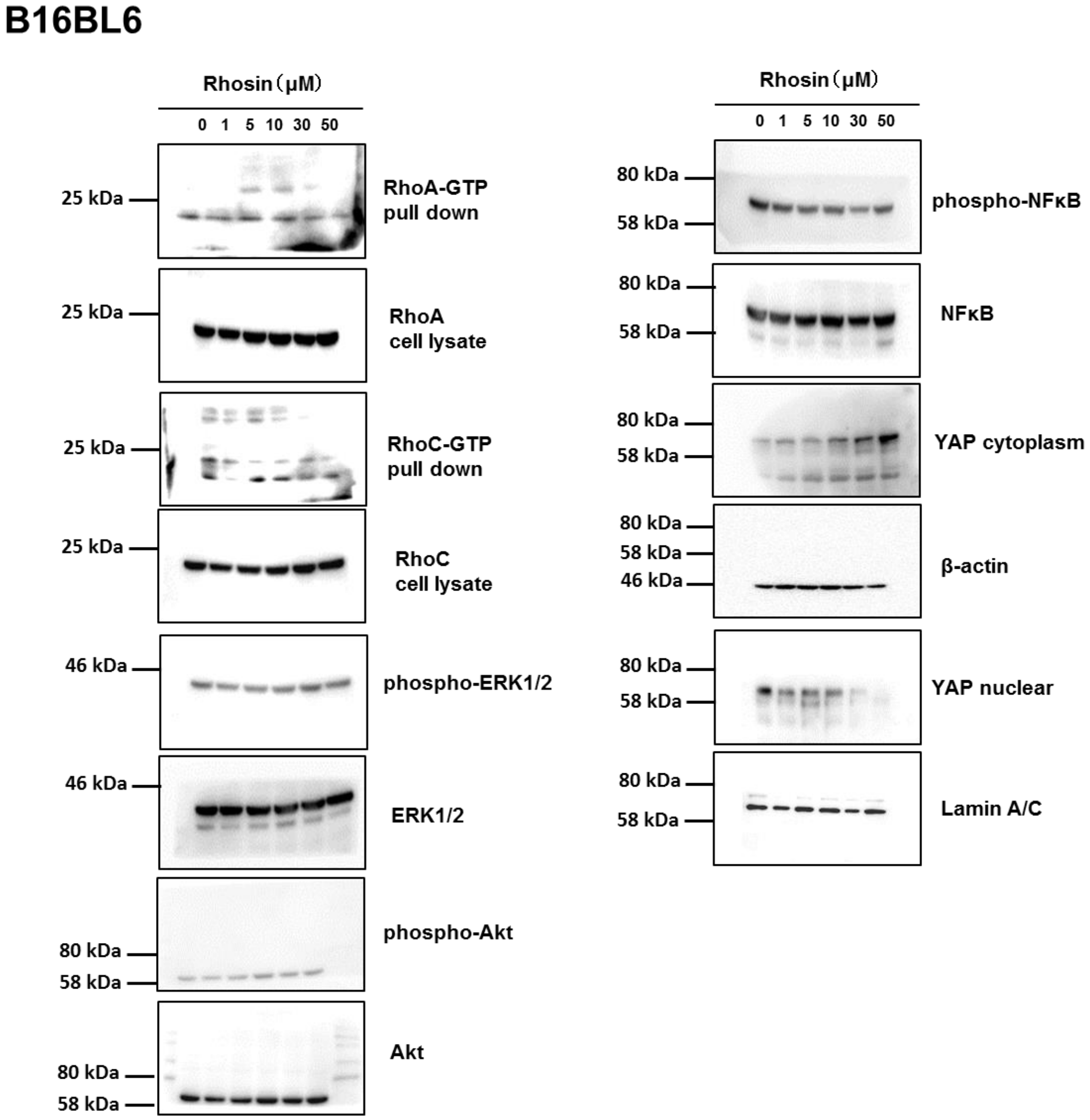
B16BL6
Rhosin (μM)
0 1 5 10 30 50
25 kDa
RhoA-GTP pull down
25 kDa
RhoA cell lysate
25 kDa
RhoC-GTP pull down
25 kDa
RhoC cell lysate
46 kDa
phospho-ERK1/2
46 kDa
ERK1/2
80 kDa
58 kDa
phospho-Akt
80 kDa
58 kDa
Akt
Rhosin (μM)
0 1 5 10 30 50
80 kDa
58 kDa
phospho-NFκB
80 kDa
58 kDa
NFκB
80 kDa
58 kDa
YAP cytoplasm
80 kDa
58 kDa
46 kDa
β-actin
80 kDa
58 kDa
YAP nuclear
80 kDa
58 kDa
Lamin A/C

4T1

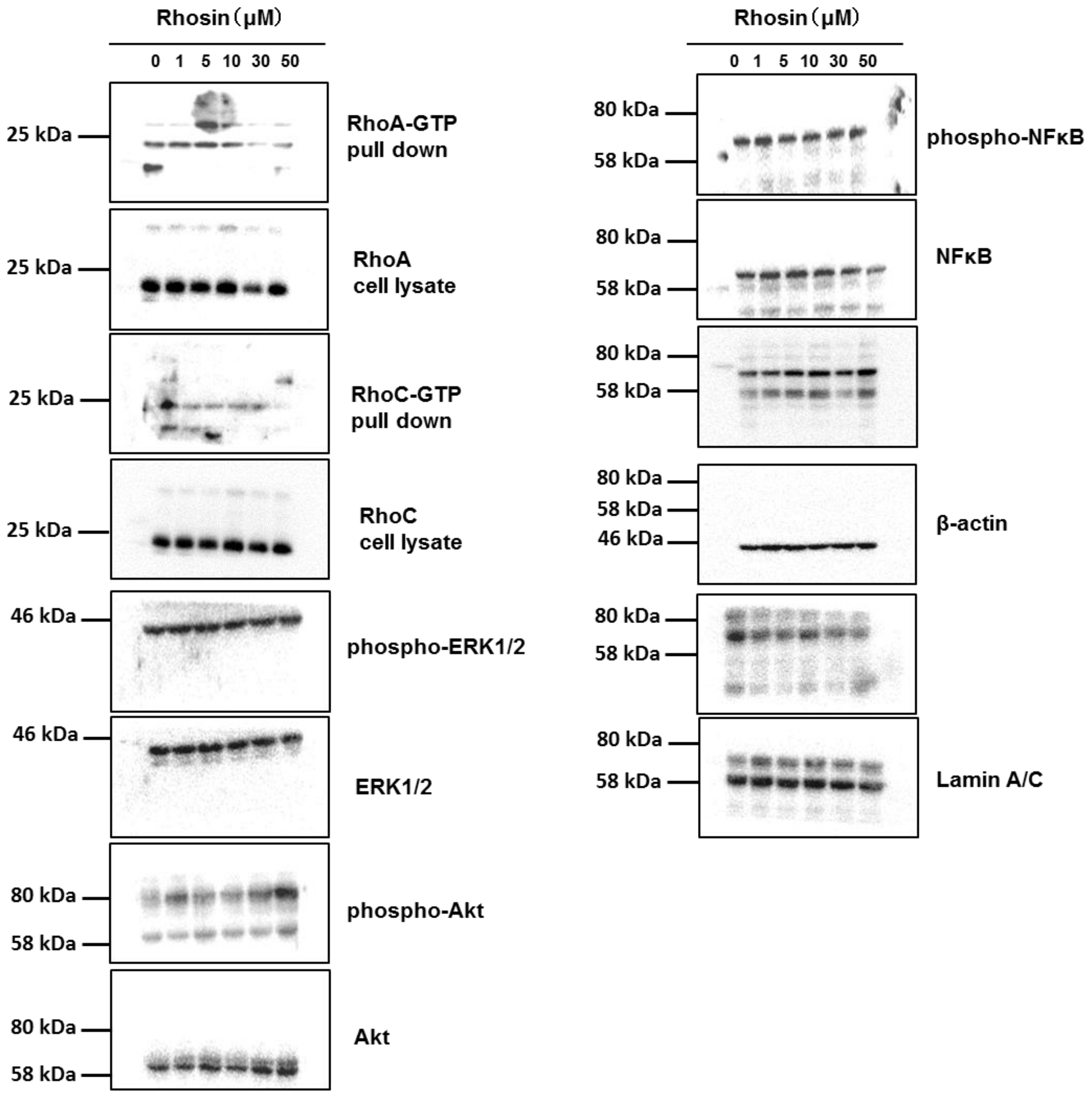

Rhosin（μM）
0 1 5 10 30 50
25 kDa
RhoA-GTP pull down
RhoA cell lysate
RhoC-GTP pull down
RhoC cell lysate
46 kDa
phospho-ERK1/2
ERK1/2
80 kDa
58 kDa
phospho-Akt
Akt
phospho-NFκB
NFκB
β-actin
Lamin A/C

Figure 2c and d

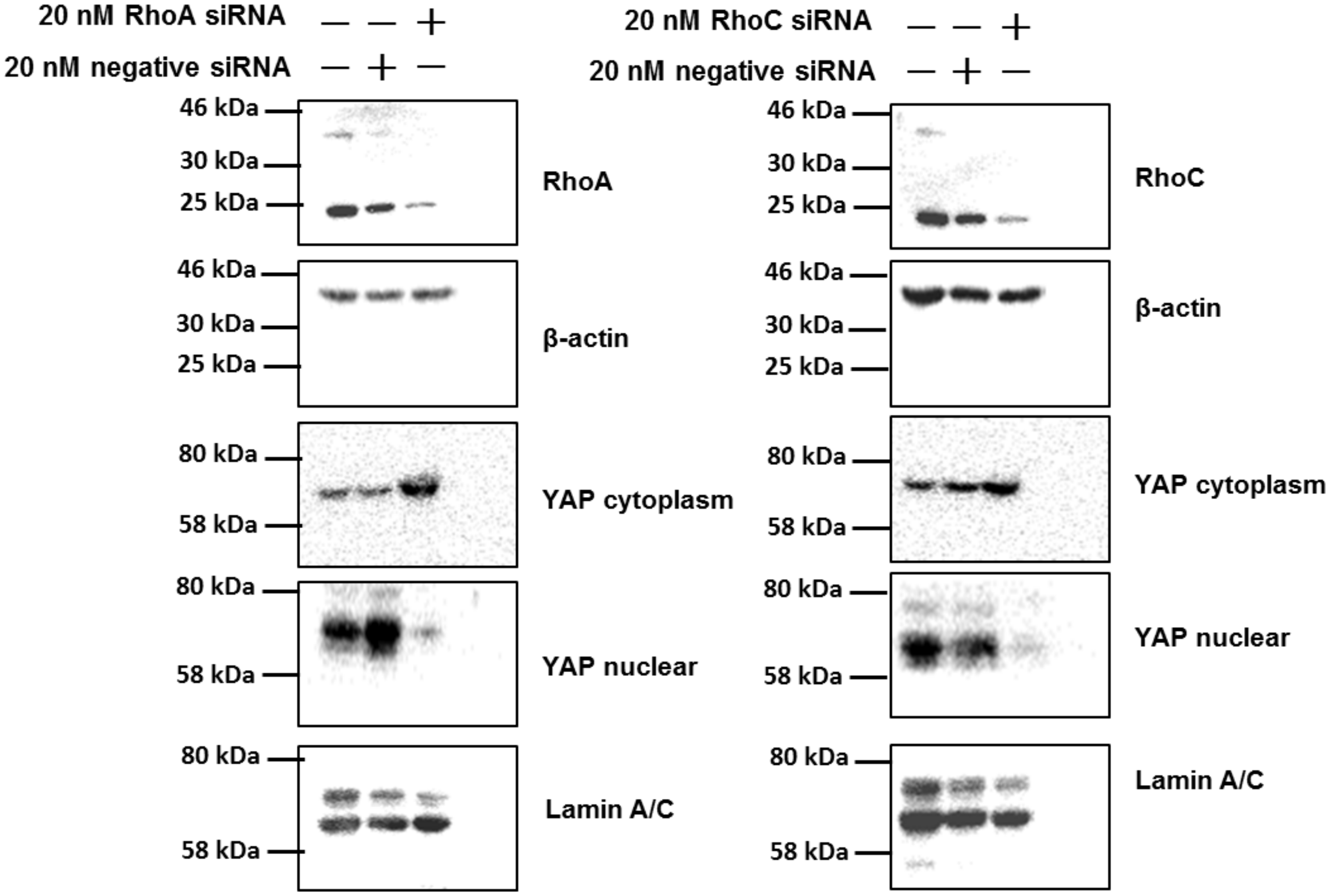

Figure 4a

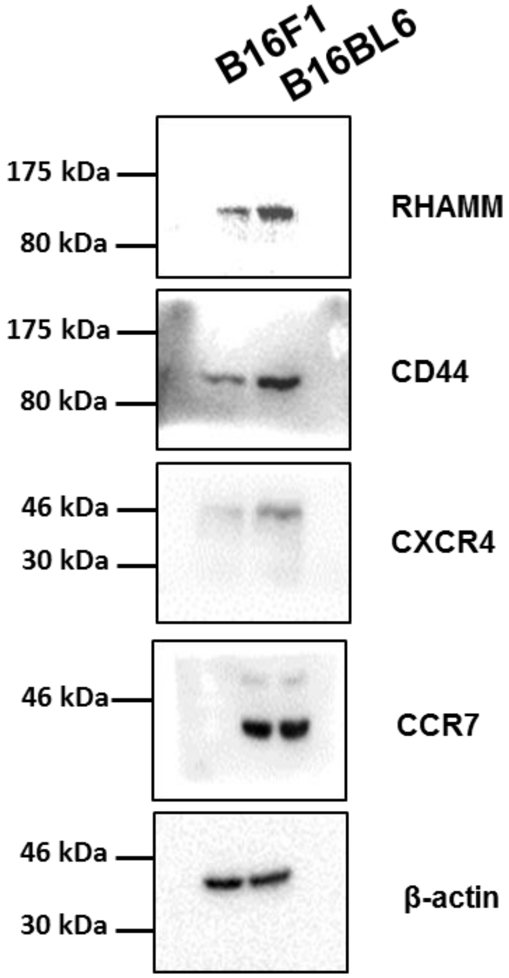

Figure 4b

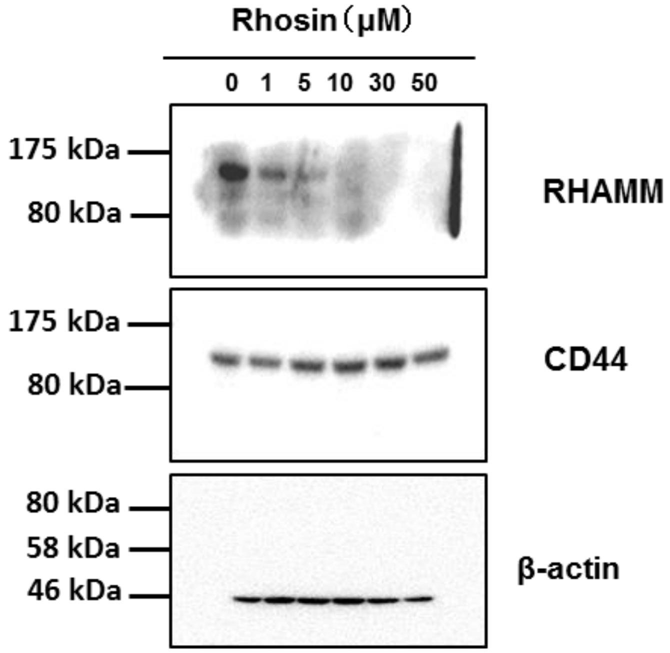

Figure 4d

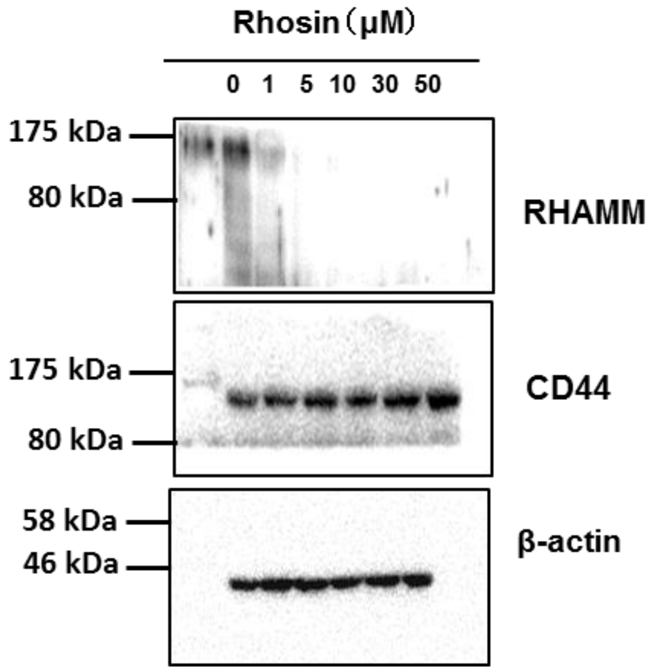

Figure 4f and g

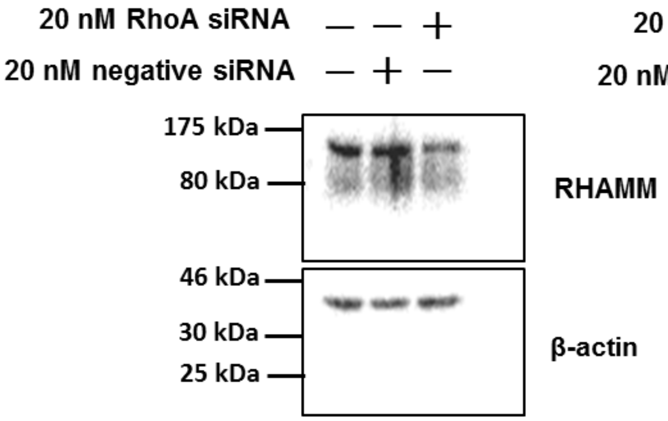

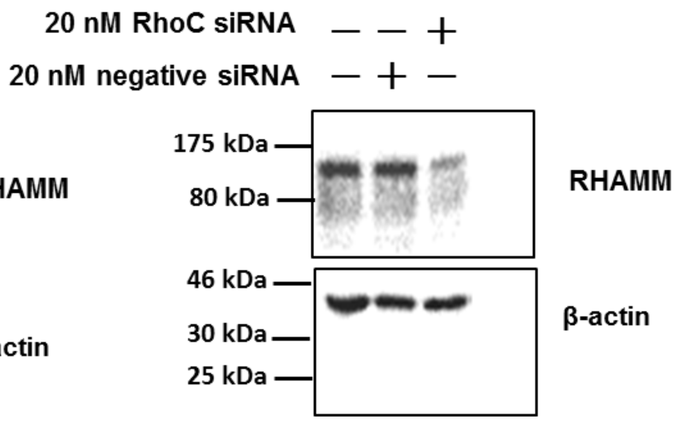

Figure 5a

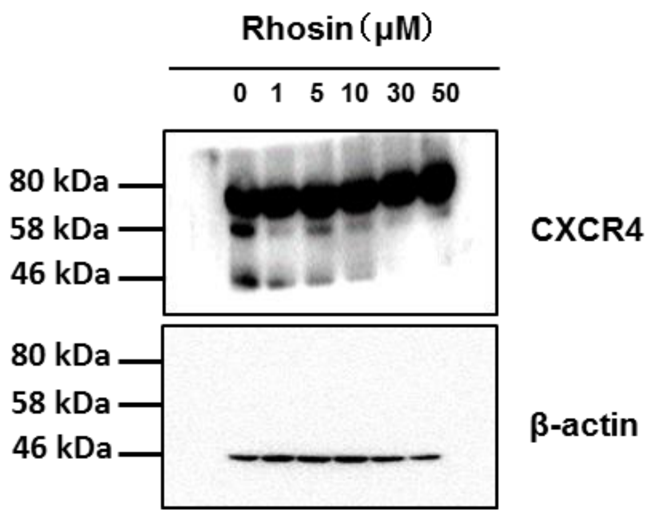

Figure 5c

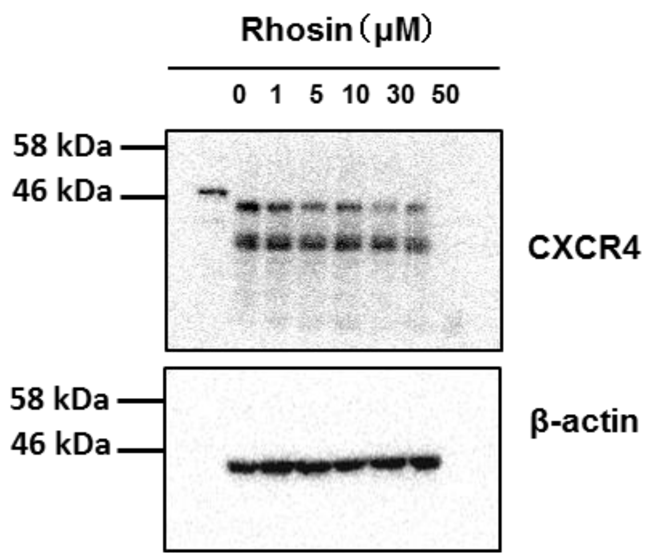

Figure 5e and f

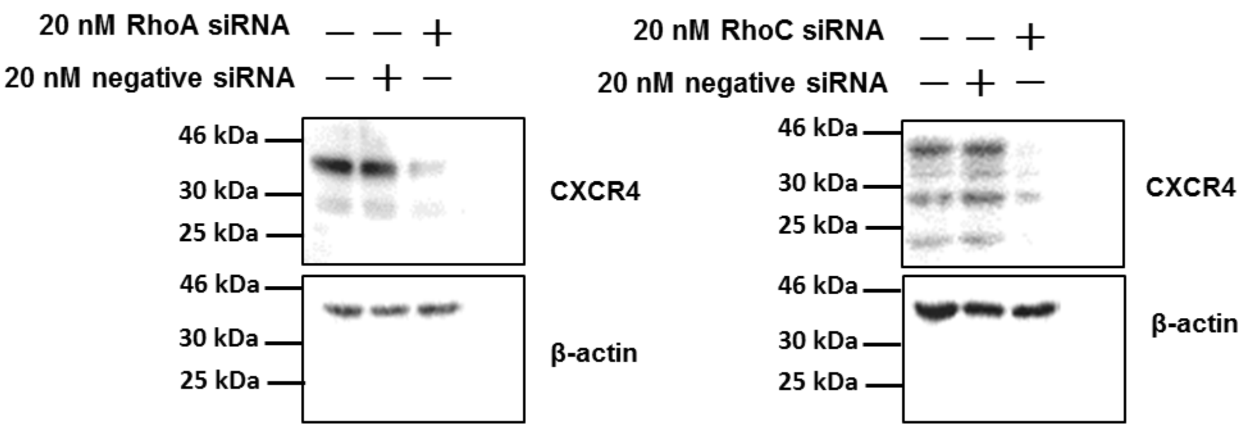

Figure 6a

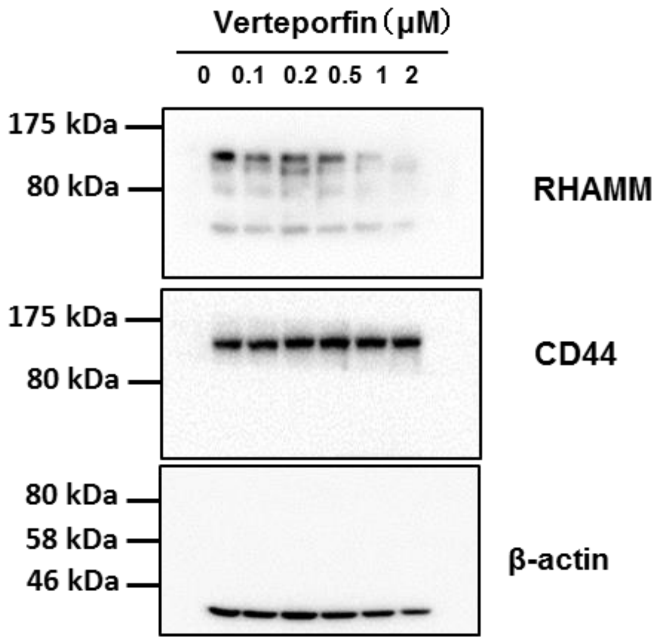

Figure 6c

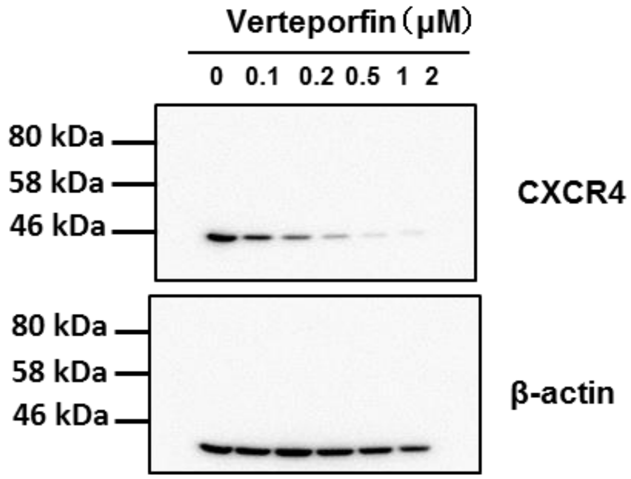

Supplementary Figure 3

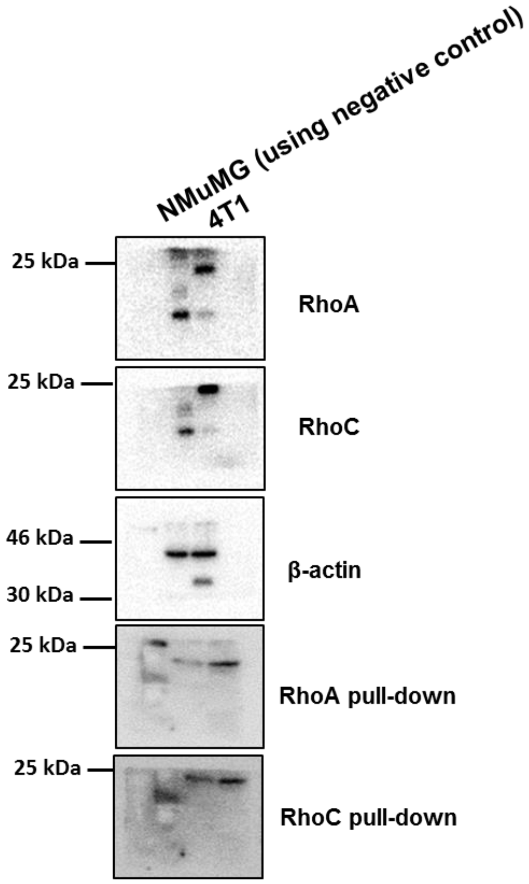

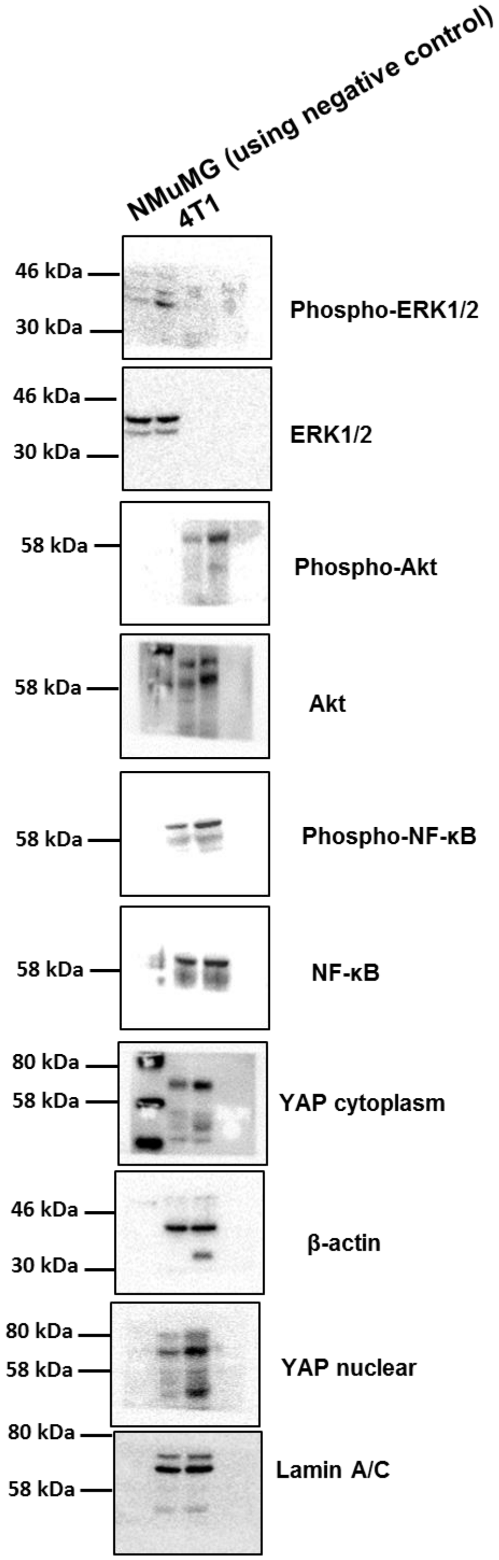

Supplement: Supplementary file 1 [file biomedicines-09-00035-s001.pdf]
